# Outcomes of hemi- vs. total arch replacement in acute type A aortic dissection: A systematic review and meta-analysis

**DOI:** 10.3389/fcvm.2022.988619

**Published:** 2022-09-27

**Authors:** Likang Ma, Tianci Chai, Xiaojie Yang, Xinghui Zhuang, Qingsong Wu, Liangwan Chen, Zhihuang Qiu

**Affiliations:** ^1^Department of Cardiovascular Surgery, Union Hospital, Fujian Medical University, Fuzhou, Fujian, China; ^2^Key Laboratory of Cardio-Thoracic Surgery (Fujian Medical University), Fujian Province University, Fuzhou, Fujian, China; ^3^Fujian Provincial Special Reserve Talents Laboratory, Fuzhou, Fujian, China; ^4^Department of Thoracic Surgery, Union Hospital, Fujian Medical University, Fuzhou, Fujian, China

**Keywords:** hemiarch replacement, total arch replacement, type A aortic dissection, meta-analysis, adult

## Abstract

**Background:**

Acute type A aortic dissections (ATAAD) pose a challenge to surgeons due to high mortality, and decision making regarding the appropriate procedure is controversial. This study compared the outcomes of hemiarch and total arch replacement for ATAAD.

**Methods:**

The PubMed, Web of Science, Embase and Cochrane databases were searched for comparative studies on hemiarch versus total arch replacement that were published before May 1, 2022.

**Results:**

We included 23 observational studies with a total of 4,576 patients. Combined data analysis showed that early mortality (RR = 0.82; 95% CI: 0.70–0.97; *P* = 0.02), incidence of postoperative permanent neurological dysfunction (RR = 0.72; 95%CI:0.54∼0.94; *P* = 0.02), and incidence of renal failure and dialysis (RR = 0.82; 95%CI:0.71∼0.96; *P* = 0.01) were all lower for hemiarch than for total arch replacement. However, hemiarch replacement had a higher rate of late mortality (RR = 1.37; 95%CI:1.10∼1.71; *P* = 0.005). There were no statistically significant differences between the two groups in terms of re-operation for bleeding, aortic re-operation, or postoperative pneumonia.

**Conclusion:**

In this study, hemiarch replacement had better early outcomes but a higher late mortality rate than total arch replacement. Decisions regarding the extent of arch repair should be made according to location and extent of ATAAD and the experience of surgeons to ensure the most favorable prognosis.

**Systematic review registration:**

[INPLASY.COM], identifier [INPLASY202250088].

## Introduction

Since acute aortic dissection was first described, it has been one of the most concerning emergent cardiac conditions owing to its high morbidity and mortality (1). Indeed, following symptom onset there is a 1–2% per hour increase in mortality rate during an ascending aortic dissection (2), which poses a great challenge for cardiac surgeons. Many patients with aortic dissection often experience a tear from the aortic intima to the arch, and the traditional surgical treatment for this has been to perform hemiarch replacement to reduce the inherent risks of surgery (3). However, this procedure may leave behind a residual false lumen in the distal aorta. Some researchers believe that total arch replacement combined with stented elephant trunk implantation is necessary for certain patients to address continued tearing of the intima to avoid subsequent reoperation (4). An intense discussion about which of the two treatments is favorable persists, as completion of the total arch replacement procedure requires personnel with rich surgical experience and expertise, and its prognosis remains relatively vague. Further, randomized controlled experiments are difficult, especially regarding ethics. Although there is an increasing trend in performing total arch replacement in patients with acute type A aortic dissections (ATAAD), controversy persists between choosing hemiarch and total arch replacement, as high-quality studies in this area are sparse. Some researchers have performed relevant meta-analyses previously (5), but newer studies have been published in the past few years, and thus these analyses require updating. In this study, we aimed to compare the early and long-term outcomes of hemiarch and total arch replacement for the treatment of ATAAD.

## Patients and methods

### Ethical statement

This study was a meta-analysis of the results of published retrospective cohort studies; therefore ethical approval and informed consent of patients were not required.

### Search strategy and selection criteria

This study protocol followed the (Preferred Reporting Items for Systematic reviews and Meta-analyses) PRISMA-P guidelines. Two authors (LKM and TCC) searched the PubMed, Web of Science, Embase, and Cochrane Central Register of Controlled Trials databases for studies published in English before May 1, 2022. They also checked the references of related literature. Our search terms were: (“DeBakey type I aortic dissection” OR “type A aortic dissection”) AND (“total aortic arch replacement” OR “aggressive arch replacement” OR “extended repair”) AND (“hemiarch replacement” OR “conservative arch replacement” OR “limited repair”). We excluded medical record reports, abstracts, expert opinions, editorial comments, review articles, and articles that could not be found in full to ensure consistency in our studies.

Two authors (LM and TC) independently determined whether the identified articles fulfilled the inclusion criteria. All included studies were required to include postoperative short-, medium-, and long-term outcomes. All studies comparing the prognosis of hemiarch and total arch replacement were included. Hemiarch replacement refers to preserving the large curved side of the aortic arch without involving the arch vessels, removing the wall of the small curved side of the blood vessels, and cutting artificial blood vessels into a slope to create an anastomosis. Total arch replacement is the replacement of the superior aortic arch as a whole or using individual branched grafts, with or without an elephant trunk stent.

Participants included patients over 18 years of age who presented with ATAAD. If an institution published multiple observational studies, the largest and most informative study with complete follow-up data was selected. Where necessary, the researchers contacted the authors of these studies to obtain any necessary information.

### Data extraction and appraisal

Data for all articles were independently collected and reviewed by two investigators (LM and TC). According to the Cochrane handbook for Systematic Reviews of Intervention, the qualities of papers were assessed independently by authors for suitability, consistency, and adequacy of study design and patient selection. For quality and bias assessment, we used the Newcastle-Ottawa Score (6) (9 = lowest risk of bias; 0 = highest risk of bias) to assess bias on three levels: selection, comparability and outcomes. The scoring was performed by two independent authors, and a score of ≥ 7 suggested no substantial bias. Finally, before the extracted data were analyzed; any discrepancies were resolved by consensus.

### Statistical analysis

For this study, we assumed clinical differences between selected studies and evaluated studies using a fixed-effects model of inverse variance. Inter-study heterogeneity was assessed using chi-square tests and the I^2^ statistic, with fixed-effects models used for data with less heterogeneity (*P* > 0.1 or *I*^2^ < 50%), and random effects models used for data with high heterogeneity.

Dichotomous data were presented in the form of risk ratios (RRs) as a summary of statistics and effect measure with 95% confidence intervals (CI). RRs were derived from the relative frequencies of the studies where available. Publication bias was assessed using funnel plots comparing log risk estimates with their standard errors. The data were synthesized using Review Manager version 5.4 (Cochrane, London, United Kingdom). The GRADEpro GDT (McMaster University and Evidence Prime, 2022. Available from gradepro.org) was used to classify the certainty of evidence (Supplementary Figure 1).

## Results

### Quantity of studies and demographics

**FIGURE 1:**
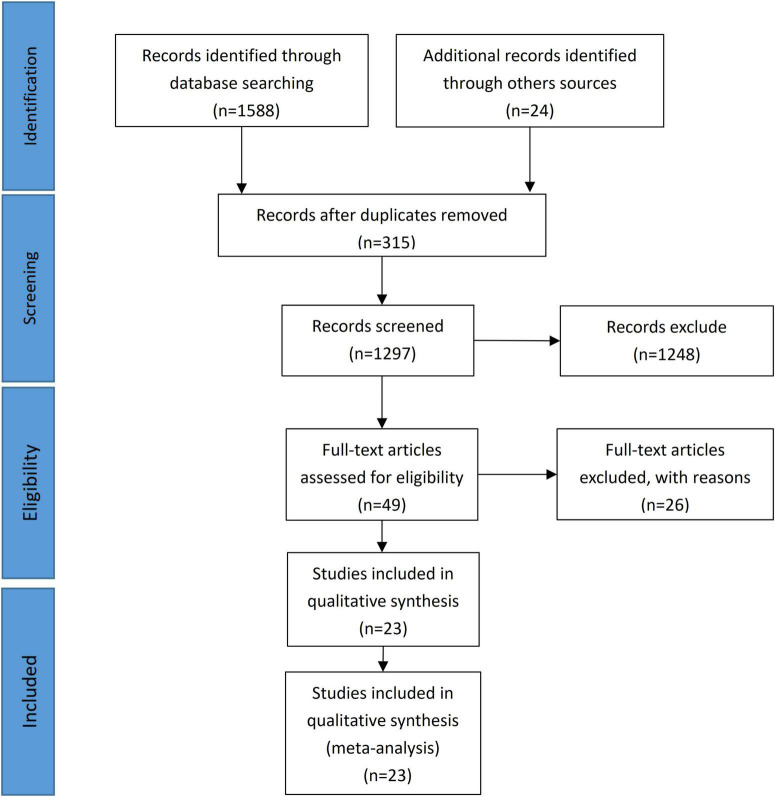
PRISMA flow diagram.

This study identified 23 retrospective observational studies that included 4,576 patients. Among them, 3,103 were hemiarch and 1,473 were total arch replacement patients. The literature selection process following the PRISMA checklist (7) is shown in Figure 1, and an overview of the selected studies is shown in Table 1. Basic patient characteristics such as age, sex, hypertension, diabetes mellitus, Marfan syndrome, renal insufficiency, and others are shown in Tables 2, 3.

**TABLE 1:** An overview of publication from selected studies.

References	Study period	Country	Number of hemiarch	Number of total arch	Total sample size	Mean follow up time	Newcastle ottawa score
Aizawa et al. (17)	2004–2014	Japan	225	42	267	57 ± 32 months	9
Chen et al. (18)	2009–2013	China	21	63	84	7.6 ± 3.4 years	7
Colli et al. (19)	1998–2015	Italy	114	21	135	5 ± 4 yesrs	7
Dai et al. (20)	2008–2010	China	41	52	93	64 ± 5.3 months	7
Di Eusanio et al. (21)	1997–2012	Italy	187	53	240	4.8 ± 3.9 years	8
Kim et al. (12)	1999–2009	Korea	144	44	188	47.5 months	9
Lee et al. (22)	2008–2018	Korea	82	16	98	48 months	8
Lio et al. (16)	2006–2013	Italy	59	33	92	30.5 months	8
Merkle et al. (23)	2006–2015	Germany	72	42	114	up to 9 years	7
Ok et al. (24)	1999–2019	Korea	248	117	265	NS	9
Omura et al. (10)	1999–2014	Japan	109	88	197	60 ± 48 months	9
Patel et al. (25)	2004–2019	USA	397	107	504	NS	7
Qin et al. (11)	2001–2015	China	41	62	103	69.6 ± 19.2 months	8
Rice et al. (26)	1999–2014	USA	440	49	489	49 months	9
Rylski et al. (27)	2001–2003	Germany	37	14	51	4.9 years 45% >5 years	9
Shi et al. (28)	2006–2011	China	71	84	155	42.7 ± 17.8 months	8
Shiono et al. (14)	1995–2005	Japan	105	29	134	up to 10 years	8
Sun et al. (4)	2003–2008	China	66	148	214	42–49 months	8
Uchida et al. (29)	1997–2008	Japan	55	65	120	67 months(3–124 months)	7
Vallabhajosyula et al. (30)	2006–2013	USA	30	31	61	60 ± 41 months	7
Vendramin et al. (31)	2006–2020	Italy	163	75	238	4.5 ± 3.5y ears	9
Yang et al. (32)	1996–2017	USA	322	150	472	5.3 years	9
Zhang et al. (15)	2002–2010	China	74	88	162	55.7 ± 33.1 months	7

NS, not specified.

**TABLE 2:** Patient’s demographics (part A).

References	Mean age		Male sex		Hypertension		Diabetes mellitus	
	HA	TA	HA	TA	HA	TA	HA	TA
Aizawa et al. (17)	66.0 ± 12.0	59.0 ± 12.0	103 (45.8%)	31 (73.8%)	NS	NS	NS	NS
Chen et al. (18)	51.0 ± 11.7	51.5 ± 10.5	9 (42.9%)	29 (46%)	NS	NS	1 (4.8%)	4 (6.3%)
Colli et al. (19)	63.0 ± 12.0	63.0 ± 13.0	49 (43%)	9 (42.9%)	61 (53.5%)	15 (71.4%)	6 (5.3%)	0 (0%)
Dai et al. (20)	49.1 ± 10.4	49.8 ± 9.6	25 (61%)	29 (55.8%)	40 (97.6%)	49 (94.2%)	1 (2.4%)	1 (1.9%)
Di Eusanio et al. (21)	64.4 ± 11.2	59.2 ± 12.3	125 (66.8%)	41 (77.4%)	138 (73.8%)	40 (75.5%)	8 (4.3%)	1 (1.9%)
Kim et al. (12)	57.6 ± 11.5	55.0 ± 12.1	69 (47.9%)	26 (59.1%)	92 (63.9%)	24 (54.5%)	6 (4.2%)	2 (4.5%)
Lee et al. (22)	60.1 ± 14.2	60.7 ± 14.3	38 (46.3%)	8 (50.0%)	45 (54.6%)	9 (56.3%)	6 (7.3%)	0 (0%)
Lio et al. (16)	66.0 ± 10.0	61.0 ± 12.0	43 (72.9%)	28 (84.8%)	51 (86.4%)	30 (90.9%)	2 (3.4%)	1 (3.0%)
Merkle et al. (23)	67.0	60.0	49 (68.1%)	27 (64.3%)	61 (84.7%)	31 (73.8%)	6 (8.3%)	1 (2.4%)
Ok et al. (24)	59.2 ± 11.9	51.9 ± 13.6	116 (46.8%)	72 (61.5%)	150 (60.5%)	62 (53.0%)	15 (6.0%)	6 (5.1%)
Omura et al. (10)	70.0 ± 11.0	61.0 ± 13.0	50 (45.9%)	62 (70.5%)	NS	NS	NS	NS
Patel et al. (25)	55.9 ± 14.1	55.3 ± 13.5	263 (66.2%)	71 (66.4%)	373 (94.0%)	97 (90.7%)	59 (14.9%)	8 (7.5%)
Qin et al. (11)	70.7 ± 3.8	69.5 ± 3.2	29 (70.7%)	42 (67.7%)	41 (100%)	62 (100%)	11 (26.8%)	15 (24.2%)
Rice et al. (26)	57.9 ± 14.8	62.4 ± 13.4	313 (71.1%)	38 (77.6%)	370 (84.1%)	40 (81.6%)	NS	NS
Rylski et al. (27)	66.0	55.0	21 (56.8%)	8 (57.1%)	31 (83.8%)	13 (92.9%)	NS	NS
Shi et al. (28)	55.9 ± 10.1	53.9 ± 12.2	53 (74.6%)	57 (67.9%)	55 (77.5%)	67 (79.8%)	12 (16.9%)	19 (22.6%)
Shiono et al. (14)	66.9 ± 13.0	59.5 ± 14.9	46 (43.8%)	15 (51.7%)	NS	NS	NS	NS
Sun et al. (4)	46.0 ± 13.0	45.0 ± 11.0	36 (54.5%)	126 (85.1%)	36 (54.5%)	107 (72.3%)	2 (3.0%)	6 (4.1%)
Uchida et al. (29)	72.3	64.4	25 (45.5%)	28 (43.1%)	NS	NS	NS	NS
Vallabhajosyula et al. (30)	58.0 ± 11.0	59 ± 12	20 (66.7%)	20 (64.5%)	26 (86.7%)	28 (90.3%)	2 (6.7%)	2 (6.5%)
Vendramin et al. (31)	69.0	58.0	106 (65.0%)	57 (76.0%)	124 (76.1%)	58 (77.3%)	11 (6.7%)	2 (2.7%)
Yang et al. (32)	61.0	57.0	226 (70.2%)	104 (69.3%)	230 (71.4%)	107 (71.3%)	21 (6.5%)	9 (6.0%)
Zhang et al. (15)	49.1 ± 12.6	45.5 ± 13.5	55 (74.3%)	74 (84.1%)	47 (63.5%)	64 (72.7%)	4 (5.4%)	4 (4.5%)

NS, not specified; HA, hemiarch; TA, total arch.

**TABLE 3:** Patient’ s demographics (part B).

References	Mafan syndrome		Renal dysfunction		Cardiogenic shock/Tamponade		Cerebrovascular accident/Stroke	
	HA	TA	HA	TA	HA	TA	HA	TA
Aizawa et al. (17)	3 (1.3%)	0 (0%)	2 (0.9%)	3 (7.1%)	38 (16.9%)	4 (9.5%)	37 (16.4%)	5 (11.9%)
Chen et al. (18)	2 (9.5%)	7 (11.1%)	0 (0%)	1 (1.6%)	NS	NS	1 (4.8%)	2 (3.2%)
Colli et al. (19)	3 (2.6%)	0 (0%)	5 (4.4%)	0 (0%)	15 (13.2%)	2 (9.5%)	31 (27.2%)	3 (14.3%)
Dai et al. (20)	2 (4.9%)	3 (5.8%)	1 (2.4%)	2 (3.8%)	2 (4.9%)	1 (1.9%)	0 (0%)	1 (1.9%)
Di Eusanio et al. (21)	5 (2.7%)	3 (5.7%)	8 (4.3%)	3 (5.7%)	25 (13.4%)	2 (3.8%)	11 (5.9%)	4 (7.5%)
Kim et al. (12)	7 (4.9%)	1 (2.3%)	NS	NS	13 (9.0%)	4 (9.1%)	4 (2.8%)	3 (6.8%)
Lee et al. (22)	4 (4.9%)	1 (6.3%)	NS	NS	NS	NS	4 (4.9%)	0 (0%)
Lio et al. (16)	NS	NS	1 (1.7%)	0 (0%)	5 (8.5%)	4 (12.1%)	NS	NS
Merkle et al. (23)	NS	NS	NS	NS	NS	NS	17 (23.6%)	13 (31.0%)
Ok et al. (24)	17 (6.9%)	12 (10.3%)	NS	NS	26 (10.5%)	12 (10.3%)	NS	NS
Omura et al. (10)	NS	NS	NS	NS	25 (22.9%)	10 (11.4%)	NS	NS
Patel et al. (25)	NS	NS	14 (3.5%)	3 (2.8%)	30 (7.6%)	2 (1.9%)	41 (10.3%)	11 (10.3%)
Qin et al. (11)	NS	NS	NS	NS	7 (17.1%)	13 (21.0%)	2 (4.9%)	2 (3.2%)
Rice et al. (26)	9 (2.0%)	1 (2.0%)	105 (23.9%)	10 (20.4%)	69 (15.7%)	9 (18.4%)	32 (7.3%)	6 (12.2%)
Rylski et al. (27)	2 (5.4%)	0 (0%)	NS	NS	3 (8.1%)	1 (7.1%)	NS	NS
Shi et al. (28)	10 (14.1%)	22 (26.2%)	5 (7.0%)	4 (4.8%)	13 (18.3%)	12 (14.3%)	2 (2.8%)	2 (2.4%)
Qin et al. (14)	5 (4.8%)	3 (10.3%)	NS	NS	49 (46.7%)	8 (27.6%)	11 (10.5%)	0 (0%)
Sun et al. (4)	5 (7.6%)	19 (12.8%)	1 (1.5%)	4 (2.7%)	8 (12.1%)	3 (2.0%)	1 (1.5%)	1 (0.7%)
Qin et al. (29)	NS	NS	5 (9.1%)	2 (3.1%)	21 (38.2%)	21 (32.3%)	12 (21.8%)	6 (9.2%)
Vallabhajosyula et al. (30)	10 (33.3%)	7 (22.6%)	3 (10.0%)	2 (6.5%)	7 (23.3%)	3 (9.7%)	1 (3.3%)	5 (16.1%)
Vendramin et al. (31)	NS	NS	16 (9.8%)	12 (16.0%)	61 (37.4%)	20 (26.7%)	28 (17.2%)	19 (25.3%)
Yang et al. (32)	16 (5.0%)	5 (3.3%)	40 (12.4%)	26 (17.3%)	67 (20.8%)	16 (10.7%)	13 (4.0%)	7 (4.7%)
Zhang et al. (15)	13 (17.6%)	21 (23.9%)	6 (8.1%)	3 (3.4%)	25 (33.8%)	17 (19.3%)	2 (2.7%)	1 (1.1%)

NS, not specified; HA, hemiarch; TA, total arch.

### Surgical technique

In patients undergoing hemiarch replacement, most of the curvature of the lesser arch curvature was excised obliquely at the aortic arch. The greater curvature of the aortic arch was left and distal anastomosis was performed. In total arch replacement, the entire arch was excised, and arteries in the upper part of the aortic arch as a whole or branches were anastomosed with the graft. Some cardiac centers implanted stents into the lumen of the distal aorta. When the patient’s aortic intimal tear was located along the ascending aorta or the lesser curvature of the arch, hemiarch replacement was chosen. If the patient’s aortic intimal tear was located at the greater curvature of the arch or involved the upper arch artery, total arch replacement was performed. For total arch replacement with a frozen elephant trunk, the stent graft was inserted into the true lumen of the thoracic aorta until it reached the predetermined position. Then the distal aorta incorporating the stent graft was securely anchored to the distal trunk of the branched prosthetic graft. The neuroprotective strategies of each study are shown in Supplementary Figure 2.

### Post-operative outcomes

#### Early mortality

**FIGURE 2:**
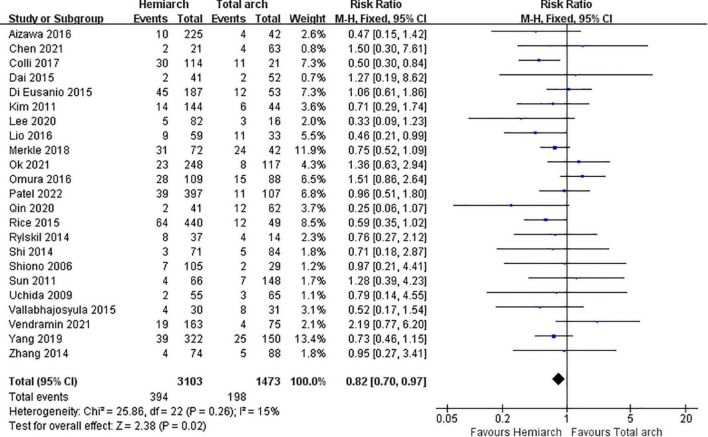
Forest plot of early mortality.

Early mortality was defined as in-hospital and 30-day mortality. A pooled analysis of 23 studies showed lower early mortality in the hemiarch replacement group (HA) compared with that in the total arch replacement group (TA), RR = 0.82; 95% CI: 0.70–0.97; *P* = 0.02; *I*^2^ = 15% (Figure 2). According to statistics, the early mortality rate of hemiarch replacement was 12.71% (3.64–43.06%), and the total arch replacement was 17.39% (3.85–57.14%) (Supplementary Figure 3).

#### Neurological dysfunction

Patients with temporary neurological dysfunction may show slurred speech, poor response to instructions, visual field defects, or localized seizures. Data from 10 studies in a pooled analysis showed no statistical differences between the two groups, RR = 0.89; 95%CI:0.69∼1.14; *P* = 0.34; *I*^2^ = 0% (Supplementary Figure 4A). Permanent neurological dysfunction may take place if coma persists or stroke occurs after surgery. Data from 16 studies were pooled to show that hemiarch replacement had a lower risk of permanent neurological dysfunction than total arch replacement, RR = 0.72; 95%CI:0.54∼0.94; *P* = 0.02; *I*^2^ = 0% (Supplementary Figure 4B).

#### Renal failure and dialysis

According to the analysis of data from 22 studies, the incidence of acute renal failure and dialysis after hemiarch replacement was significantly lower, RR = 0.82; 95%CI:0.71∼0.96; *P* = 0.01; *I*^2^ = 0% (Supplementary Figure 4C).

#### Re-operation

There were 18 and 16 studies reporting re-operation for bleeding and aortic re-operation (proximal and distal). The analysis of the data showed no significant differences between hemiarch and total arch replacement regarding re-operation, RR = 0.89; 95% CI:0.72∼1.11; *P* = 0.30; *I*^2^ = 0% (Supplementary Figure 4D), and RR = 1.11; 95% CI:0.87∼1.41; *P* = 0.41; *I*^2^ = 17% (Supplementary Figure 4E), respectively.

#### Pneumonia

There were 10 studies that recorded the postoperative incidence of pneumonia in patients, and the aggregate data analysis found no significant statistical differences between the two groups, RR = 0.67; 95% CI:0.44∼1.04; *P* = 0.08; *I*^2^ = 60% (Supplementary Figure 4F).

#### Late mortality

**FIGURE 3:**
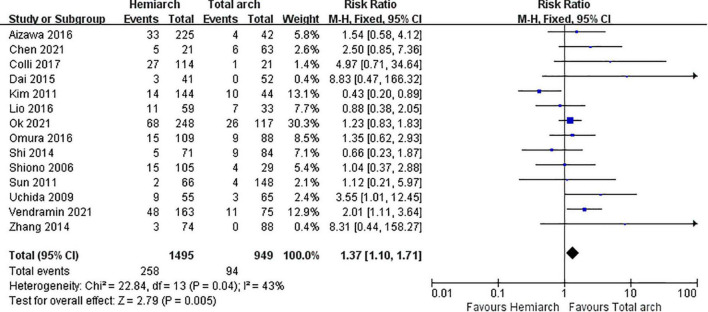
Forest plot of late mortality.

Late mortality was defined as patient death that occurred during follow-up. Summarizing the data of 14 studies, we found that the late mortality of hemiarch replacement was higher than total arch replacement, RR = 1.37; 95% CI:1.10∼1.71; *P* = 0.005; *I*^2^ = 43% (Figure 3).

#### Publication bias

Funnel plots revealed no evidence of publication bias regarding early mortality (Supplementary Figure 5A), temporary neurological dysfunction, permanent neurological dysfunction, renal failure and dialysis, re-operation for bleeding, aortic re-operation, or late mortality. However, the funnel plots did suggest some publication bias regarding pneumonia (Supplementary Figure 5B). Thus, we used the trim-and-fill method to adjust the analysis, which did not significantly alter the findings.

### Comment

In this study, results showed the incidences of early mortality, postoperative permanent neurological dysfunction, renal failure, and dialysis of hemiarch replacement to be lower than those in total arch replacement. However, the late mortality of hemiarch replacement was higher than that in total arch replacement, which differed from a previous study (5). We included up-to-date data in the English literature (Last Date access time as of May 2022). This may have been the reason for this difference.

Until recently, ATAAD was considered a severe and fatal disease. Thus, the selection of surgical strategy should be based not only on the general condition of patients and the extent of the dissection, but also the experience of cardiovascular surgeons. Cardiovascular surgeons have different views on the scope of arch surgeries for this condition. Ohtsubo (8), Westaby (9) and their colleagues have shown better early and late outcomes for hemiarch replacement. However, the results of Sun et al. (4) and Omura et al. (10) showed the ability for total arch replacement to completely repair the aorta and reduce the possibility of subsequent re-operation. In contrast, a recent study by Qin and his colleagues showed total arch replacement to have a higher mortality and surgical risk than hemiarch, and although the total arch replacement had reduced adverse arterial events later on, there was no difference in 5-year survival between the two groups (82.5 ± 60% vs. 75.2 ± 5.6%, *P* = 0.151) (11). These findings were based on data from their local hospitals, which inevitably carries the limitation of small sample size; further, differences in technique among surgical operators also has an impact on patient outcomes. In this analysis, data was pooled from 23 studies with a total of 4,576 patients from different countries, with the intent of eliminating these limitations and providing more credible findings.

Our results showed lower early mortality for hemiarch replacement, while there was no significant difference between the two procedures for re-operation rate. For emergency patients with aortic dissection, survival is the first priority, and thus the attending physician should treat the patient in the shortest possible time to avoid pericardial tamponade or rupture of the aorta, leading to death. Therefore, we believe that surgeons may perform hemiarch replacement in patients with intima tears in the root, the ascending aorta, or the proximal aorta to ensure greater chance of survival.

In addition, total arch replacement showed higher incidence of permanent neurological dysfunction, kidney failure, and dialysis in this study, which corresponds with previous reports (5, 12). In total arch replacement, longer cardiopulmonary bypass time, aortic cross-clamping time, and the selective antegrade cerebral perfusion time were was associated with ischemia reperfusion injury of the organs. Further, when the elephant trunk stent was inserted into the true lumen of the descending aorta during the total arch replacement procedure, the risk of paraplegia and spinal cord injury increased.

However, our findings also suggested that the hemiarch group had higher late mortality during follow-up, as any residual dissection may increase the risk of rupture or reoperation. In line with this finding, Omura et al. (10) reported that the incidence of distal aortic events may be lower in patients following total arch replacement, and Yoshitake et al. (13) reported that patients that underwent total arch replacement with a frozen elephant trunk stent performed better in long-term survival. Overall, total arch replacement can better repair the aorta, increase false lumen thrombosis, and reduce later aortic adverse events. Therefore, total arch replacement was a more favorable treatment choice for patients with intimal tears involving the large curvature and the thoracic-abdominal descending aorta.

For patients with Marfan syndrome, a total arch replacement may also be a better decision. Studies have shown Marfan syndrome as a statistically significant risk factor for re-operation (14), and Zhang et al. (15) has suggested that extensive arch replacement should be more actively used due to a weak aortic wall in patients with Marfan syndrome. Further, Lio et al. (16) also posited that total arch replacement or more aggressive approaches may be preferred in young patients and those with Marfan syndrome. Our research also showed that there was no significant difference in the rate of re-operation between hemiarch and total arch replacement, but total arch replacement did carry the advantage of lower late mortality. Therefore, it was more appropriate for younger patients and patients with Marfan syndrome to choose total arch replacement.

It must be stated that meta-analysis was a double-edged weapon. Meta-analysis was a reanalysis of existing research results, and its data source was limited by many factors, such as publication bias and small sample size. Moreover, some diseases were difficult to conduct randomized controlled trials, and the demonstration strength of meta-analysis was far less than that of large-scale randomized controlled trials. The lack of homogeneity among the selected studies is always a threat to the consistency of the conclusions. However, meta-analysis can analyze the differences and reasons of multiple similar studies. When the results of multiple clinical trials are inconsistent or divergent, more scientific conclusions can be drawn through combined analysis. Our research results were also ideal and may provide some guidance for the treatment of ATAAD in the future.

### Limitations

Our study had several limitations. First, we were unable to guarantee that patients included in the study had the same baseline level, and inherent differences between patients could not be avoided. Second, we were unable to guarantee that the same surgical techniques existed in all centers, which may have affected the statistics for patient outcomes. Regarding multiple surgical procedures for total arch replacement, we did not perform a subgroup analysis to count prognostic outcomes for the different surgical procedures. There was also a treatment bias in different hospitals, as total arch replacement was more often used in younger patients and in patients with Marfan syndrome, and we struggled to remove this bias. Finally, patient loss to follow-up and unreported deaths and complications had an impact on the pooled statistical results. As healthcare advances, it may be possible that subsequent studies will yield different outcomes.

## Conclusion

In conclusion, we found that hemiarch replacement had a lower incidence of early mortality, postoperative permanent neurological dysfunction, renal failure, and dialysis in patients with AAAD. However, the late survival of the total arch replacement was better than that for hemiarch replacement. As ATAAD can be a dangerous emergent condition, we believe that early survival is also important. For aortic intimal tears confined to the root, the ascending aorta, or the proximal aorta, hemiarch replacement was more desirable. When intimal tears involved the arch and the thoracic-abdominal descending aorta, total arch replacement were more appropriate, and may allow patients to obtain better long-term results. Surgical strategy was also based on the experience of surgeons. These findings should provide some guidance on choosing an appropriate approach for the treatment of ATAAD.

## Data availability statement

The original contributions presented in this study are included in the article/Supplementary material, further inquiries can be directed to the corresponding authors.

## Author contributions

LM and ZQ contributed to conception and design of the study. LM and TC organized the database. LM performed the statistical analysis and wrote the first draft of the manuscript. XZ and QW wrote sections of the manuscript. LC contributed to supervision. ZQ revised the manuscript critically for important intellectual content. All authors contributed to manuscript revision, read, and approved the submitted version.

## Supplementary material

Supplementary Figure 1 GRADEpro GDT.

Supplementary Figure 2 Neuroprotection strategies in hemiarch and total arch replacement.

Supplementary Figure 3 Early mortality.

Supplementary Figure 4 Forest plots. **(A)** Forest plot of temporary neurological dysfunction. **(B)** Forest plot of permanent neurological dysfunction. **(C)**. Forest plot of renal failure and dialysis. **(D)** Forest plot of re-operation for bleeding. **(E)** Forest plot of aortic re-operation. **(F)** Forest plot of pneumonia.

Supplementary Figure 5 Funnel plots. **(A)** Funnel plot of early mortality. **(B)** Funnel plot of pneumonia.
