# Supplementary figures and images for: Outcomes of hemi- vs. total arch replacement in acute type A aortic dissection: A systematic review and meta-analysis
Source: Front Cardiovasc Med. 2022 Sep 27;9:988619. doi: 10.3389/fcvm.2022.988619 (PMC9552831; doi:10.3389/fcvm.2022.988619)

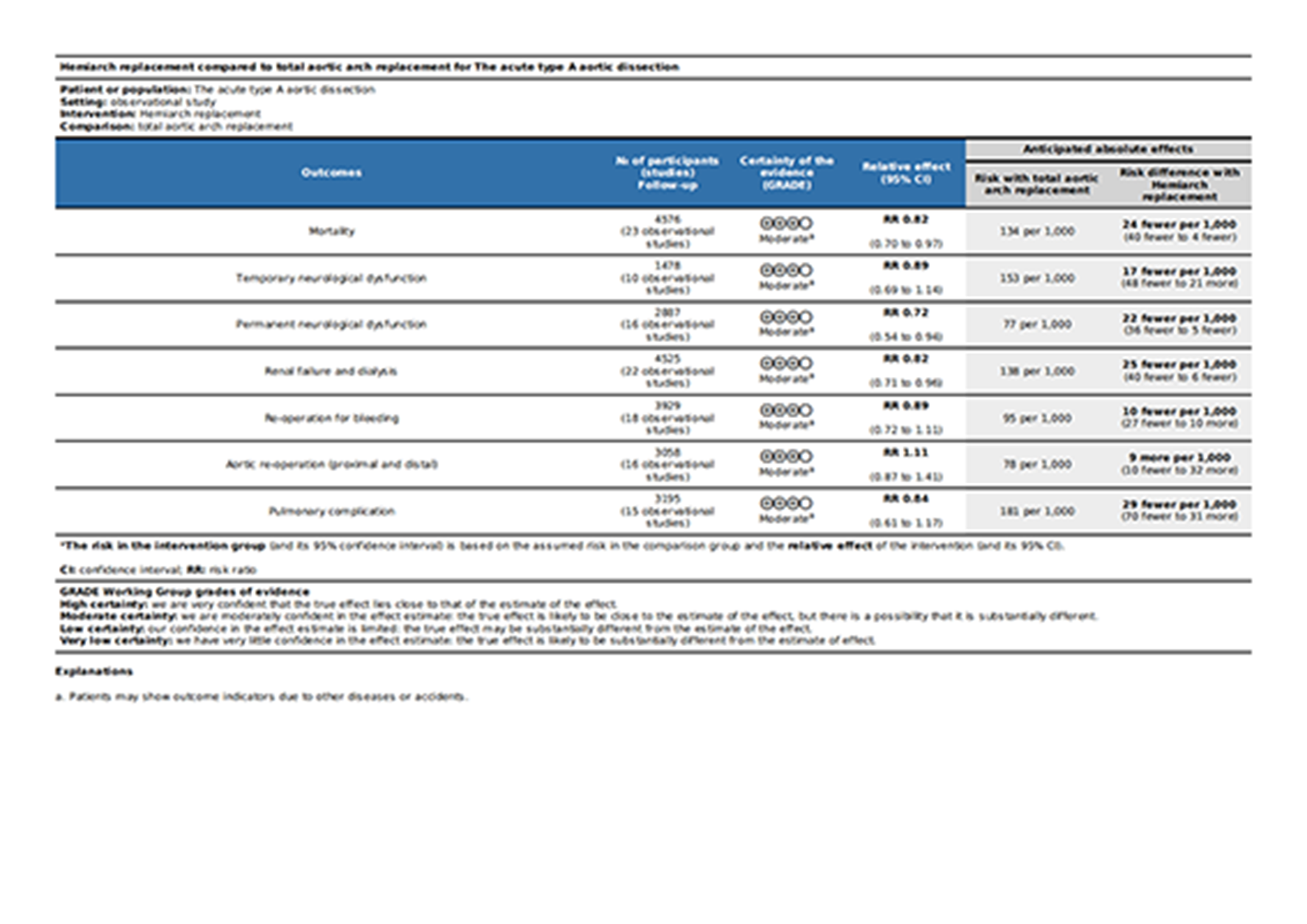

Supplement: Supplementary Figure 1 — GRADEpro GDT. [file Image_1.TIF]

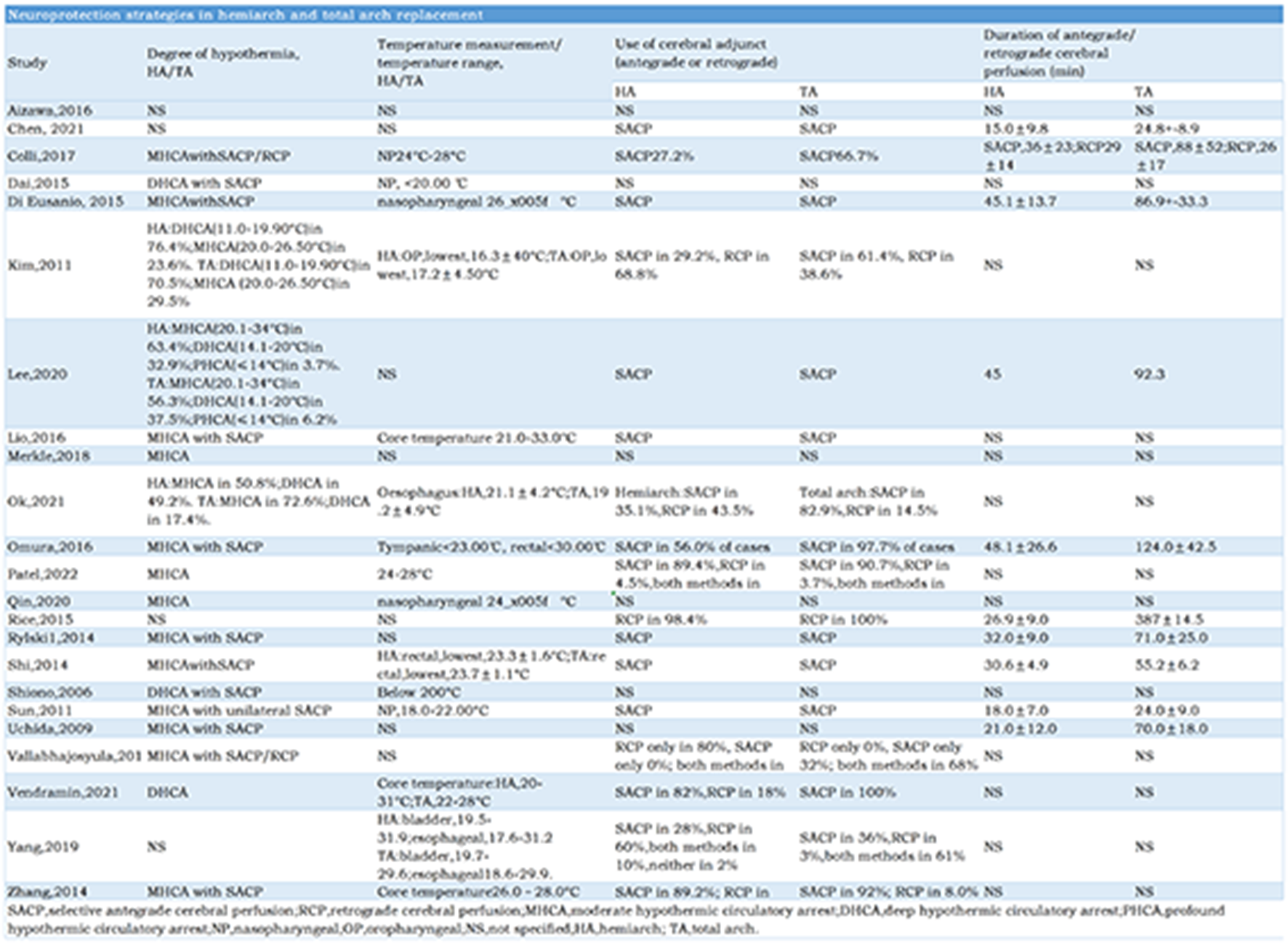

Supplement: Supplementary Figure 2 — Neuroprotection strategies in hemiarch and total arch replacement. [file Image_2.TIF]

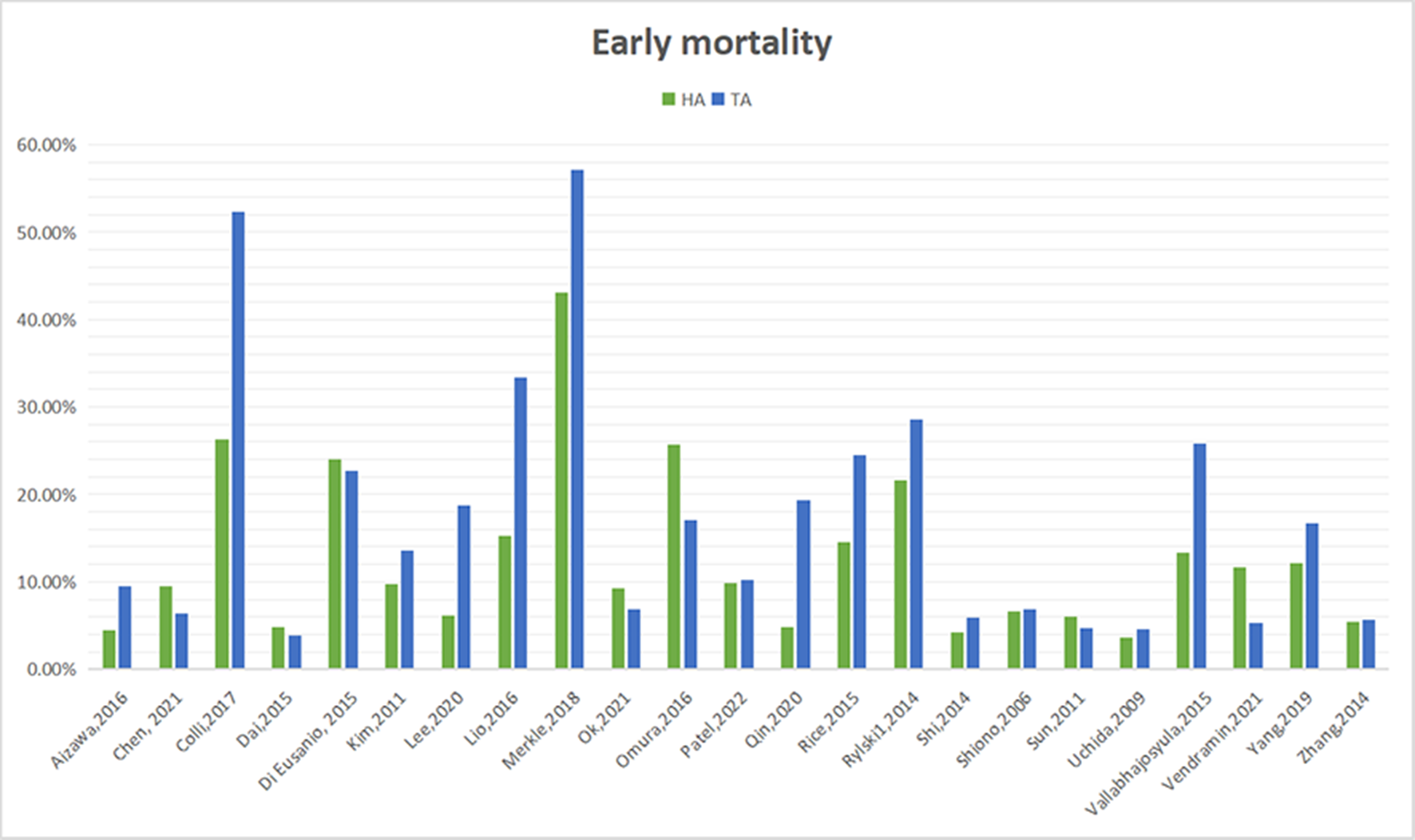

Supplement: Supplementary Figure 3 — Early mortality. [file Image_3.tif]

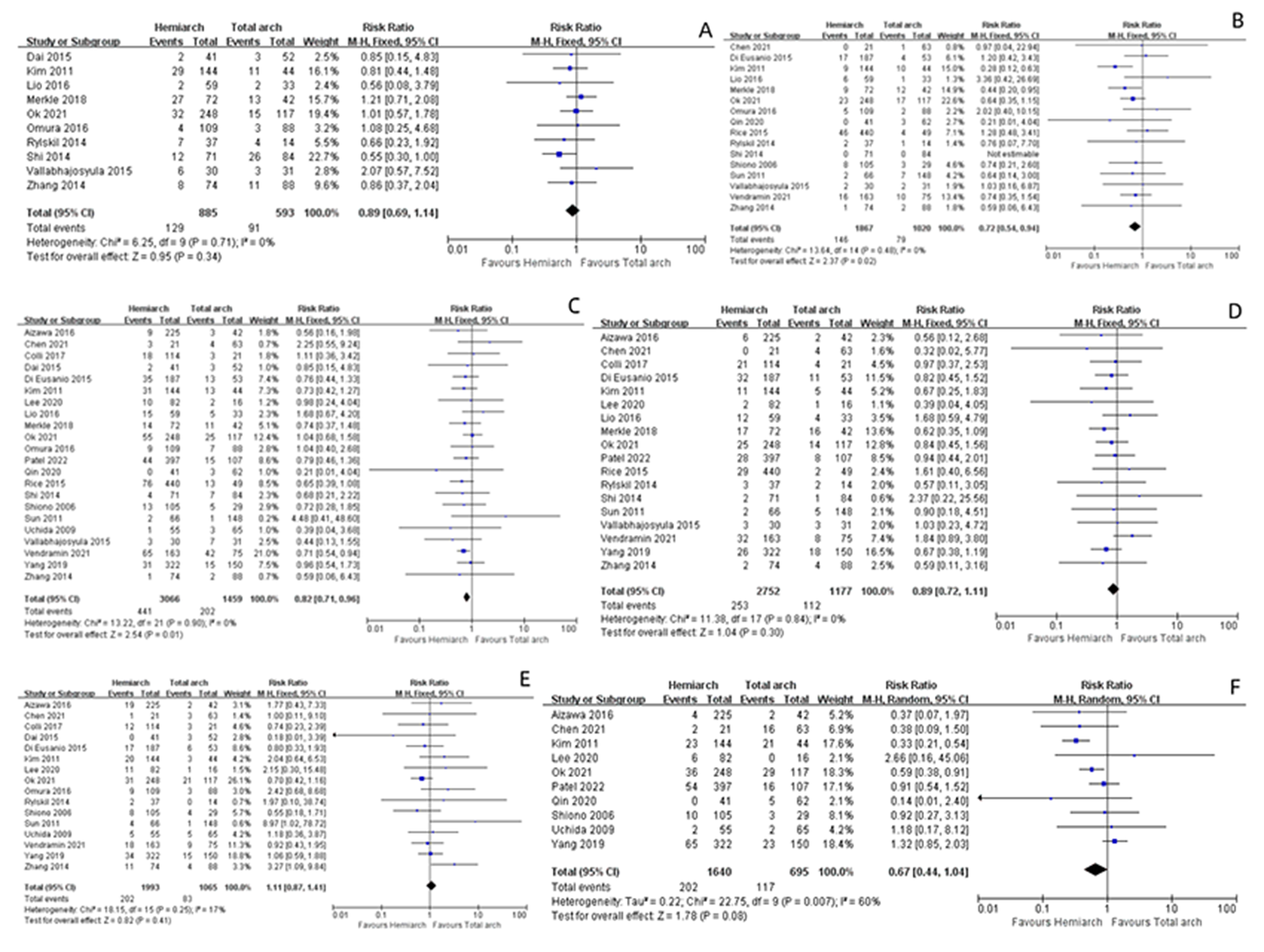

Supplement: Supplementary Figure 4 — Forest plots. (A) Forest plot of temporary neurological dysfunction. (B) Forest plot of permanent neurological dysfunction. (C). Forest plot of renal failure and dialysis. (D) Forest plot of re-operation for bleeding. (E) Forest plot of aortic re-operation. (F) Forest plot of pneumonia. [file Image_4.tif]

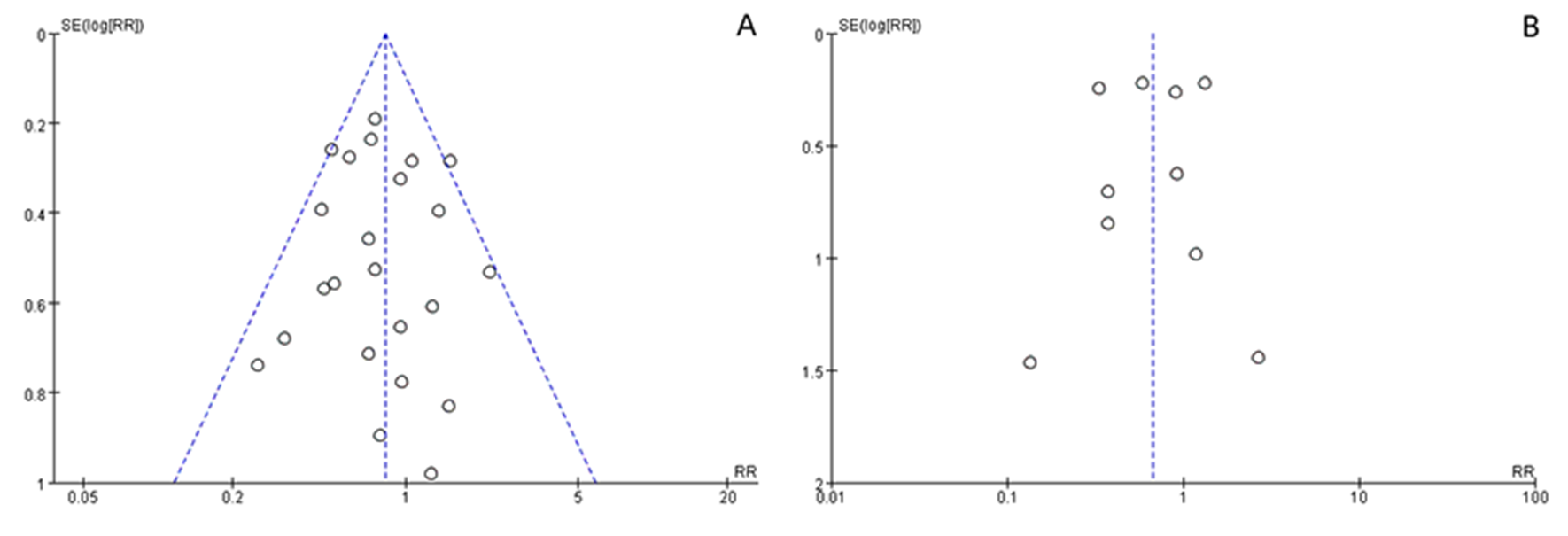

Supplement: Supplementary Figure 5 — Funnel plots. (A) Funnel plot of early mortality. (B) Funnel plot of pneumonia. [file Image_5.tif]
